# Complete instars’ morphology of *Zercon forsslundi* Sellnick, 1958 (Parasitiformes: Mesostigmata) with notes on distribution and evolution

**DOI:** 10.1038/s41598-025-06732-0

**Published:** 2025-07-02

**Authors:** Sławomir Kaczmarek, Tomasz Marquardt, Snorre B. Hagen, Cornelya F. C. Klutsch, Steffen Roth, Anna Seniczak

**Affiliations:** 1 Department of Evolutionary Biology, Faculty of Biological Sciences, Kazimierz Wielki University, Bydgoszcz, Poland; 2 NIBIO - Division of Environment and Natural Resources, Norwegian Institute of Bioeconomy Research, Svanhovd, Norway; 3 Department of Natural History, University Museum of Bergen, University of Bergen, Bergen, Norway; 4 Faculty of Applied Ecology, Agricultural Sciences and Biotechnology, Inland Norway University of Applied Sciences, Elverum, Norway

**Keywords:** Biogeography, Glacial relicts, Mites, Ontogeny, Speciation processes, Zerconidae, Biodiversity, Biogeography, Taxonomy, Zoology

## Abstract

To date, only adults of *Zercon forsslundi* have been known. According to the description of this species, it has been the first report of *Zercon* with clear differences in opisthonotal chaetotaxy between females and males, a unique character in the genus. At the same time, *Z. forsslundi* belongs to a unique group of *Zercon* species with dorsal setae *J5* in females clearly longer than setae *J1***–***J4*, the latter ones being short and of similar length. Our description is the first-ever report on the full morphological ontogeny of *Z. forsslundi*, the mite species, the adult which is the most similar to the recently described *Zercon hamaricus* and to a lesser extent to *Zercon polonicus*. The morphological closeness of the above-mentioned species has been confirmed in immature stages. We also studied the geographic distribution of *Z. forsslundi* and its above-mentioned congeners with remarks on their evolutionary affinity on the background of dispersal abilities and faunal dispersal after the Last Glacial Maximum. We also report the occurrence of *Z. forsslundi* and *Z. hamaricus* in the Northern Norway for the first time. Ranges of *Z. forsslundi* and *Z. hamaricus* overlap in the area of N Norway. Also, the microenvironmental sympatry was confirmed for these taxa. We also proved the niche overlap of these two species, which is probably limited to the northern verge of Eurasia. *Zercon polonicus*, not present in the current Arctic fauna, probably is a post-glacial relict, inhabiting more southern mountainous areas of Europe (Carpathians and Alps).

**Supplementary Information:**

The online version contains supplementary material available at 10.1038/s41598-025-06732-0.

## Introduction

Until now, nearly 500 species of *Zercon* have been described, mostly inhabiting the Northern Hemisphere and the Holarctic Realm, however, some species have also been found on the Central Mexican Plateau (Neotropical realm) representing the southernmost occurrence of the family^1–3^. So far, only the adults of *Zercon forsslundi* Sellnick, 1958^4^ have been described. The separation of males and females of *Z. forsslundi* in the Sellnick’s^4^ key resulted from the peculiar sexual dimorphism of this species. Males and females of this species differ in opisthonotal chaetotaxy, which is unusual in the genus^5^. This peculiar character is partly confirmed in the group of ten *Zercon* species with females possessing dorsal setae *J5* clearly longer when compared with short and similar in length setae *J1***–***J4*. Kaczmarek et al.^5^ distinguished two subgroups of these species, based on morphology of setae *S1*–*S3*, *r4*–*r5*–*s6* and *R1*–*R6*. In the first subgroup, there are the Palearctic *Z. forsslundi*, *Z. hamaricus* Kaczmarek et al., 2021^5^ and *Z. polonicus* Błaszak, 1970^6^. The second subgroup is comprised of Nearctic *Z. canadensis* Halašková, 1977^7^, *Z. carolinensis* Halašková, 1969^8^, *Z. columbianus* Berlese, 1910^9^, *Z. fenestralis* Evans, 1955^10^, *Z. lindquisti* Halašková, 1977^7^, *Z. lucidus* Sikora, 2014^11^, and Neotropical *Z. mexicanus* Ujvári, 2011^1^; the latter one representing the southernmost occurrence of the family and the only known occurrence of Zerconidae outside the Holarctic.

The similarity between *Z. forsslundi*, *Z. polonicus* and *Z. hamaricus* was proved by Kaczmarek et al.^5^. Here, we provide a description of all the ontogenetic stages of *Z. forsslundi* expanding the morphological comparison with its most similar congeners. We also widen zoogeographical data and discuss the distribution of *Z. forsslundi* and closely related taxa, hypothesizing their evolutionary connections.

## Material and methods

Individuals of *Zercon forsslundi* (det. S. Kaczmarek) used in present description were collected on June 5, 2018, by Steffen Roth in Spitsbergen, Svalbard, Norway from two sites: Longyearbyen, Bjørndalen (78.20371 N, 15.31903 E); Kapp Linne, near Isfjord Radio Station (78.03989 N, 13.6457 E). New localities of occurrence of *Z. forsslundi* and *Z. hamaricus* in the Northern Norway are listed in Table A1 (see Additional material).

**Table 1:** Length (mean ± SD/range in µm) and smoothness of podonotal setae in females, males and juveniles of *Zercon forsslundi*.

	♀♀		♂♂		D		P		L	
	Length	Type	Length	Type	Length	Type	Length	Type	Length	Type
	*Z. forsslundi*									
*j1*	38 ± 1/36–41	B	32 ± 2/29–35	B	26 ± 1/24–28	B	25 ± 1/24–26	B	22 ± 1/20–25	B
*j2*	21 ± 1/20–23	S	16 ± 1/16–19	S	16 ± 2/12–19	S	13 ± 1/11–15	S	nd	–
*j3*	20 ± 1/20–23	S	18 ± 2/15–21	S	19 ± 1/18–22	S	20 ± 1/19–22	S	18 ± 1/16–19	B
*j4*	25 ± 1/23–27	S	20 ± 2/18–23	S	20 ± 1/19–23	S	20 ± 1/19–21	S	19 ± 1/18–20	B
*j5*	23 ± 2/21–26	S	20 ± 1/18–22	S	16 ± 1/14–19	S	18 ± 1/17–19	S	16 ± 2/14–19	B
*j6*	26 ± 1/24–28	S	20 ± 1/18–21	S	17 ± 1/15–19	S	18 ± 1/17–20	S	22 ± 2/20–24	B
*z1*	nd in *Zercon*									
*z2*	21 ± 1/18–24	S	16 ± 1/14–19	S	17 ± 1/15–18	S	20 ± 1/19–22	S	18 ± 1/16–21	S
*z3*	25 ± 1/22–28	S	20 ± 1/18–22	S	21 ± 1/18–22	S	nd	–	nd	–
*z4*	28 ± 1/27–31	S	24 ± 2/21–26	S	26 ± 2/21–29	S	30 ± 1/29–32	S	36 ± 3/31–41	B
*z5*	21 ± 2/17–24	S	19 ± 1/17–22	S	17 ± 1/14–19	S	15 ± 1/14–17	S	16 ± 2/13–20	S
*z6*	22 ± 1/20–24	S	17 ± 1/15–19	S	11 ± 1/10–13	S	nd	–	nd	–
*s1*	15 ± 1/14–17	S	13 ± 2/10–15	S	10 ± 2/9–14	S	nd	–	nd	–
*s2*	21 ± 2/18–24	S	17 ± 2/14–20	S	14 ± 2/12–18	S	nd	–	nd	–
*s3*	44 ± 2/40–48	B	38 ± 1/37–39	B	42 ± 1/41–44	B	nd	–	nd	–
*s4*	26 ± 1/24–29	S	21 ± 1/19–22	S	20 ± 1/18–21	S	23 ± 2/18–26	S	48 ± 2/44–51	B
*s5*	28 ± 1/26–30	S	23 ± 1/21–24	S	24 ± 2/23–28	S	31 ± 1/30–34	S	nd	–
*s6*	38 ± 2/33–41	B	31 ± 2/27–34	B	34 ± 2/31–38	B	24 ± 3/20–28	B	5 ± 0.4/4–5	S
*r1*	19 ± 1/16–21	S	13 ± 1/13–15	S	11 ± 1/9–13	S	nd	–	nd	–
*r2*	25 ± 2/19–28	S	21 ± 1/19–22	S	16 ± 1/15–18	S	42 ± 2/40–46	B	nd	–
*r3*	49 ± 2/45–52	B	39 ± 1/38–40	B	33 ± 2/31–38	B	31 ± 1/30–32	B	nd	–
*r4*	24 ± 2/19–28	B	19 ± 2/16–21	B	13 ± 1/12–14	B	nd	–	nd	–
*r5*	31 ± 2/27–34	B	24 ± 2/21–27	B	21 ± 3/15–26	B	10 ± 1/8–12	S	nd	–

D – deutonymph, P – protonymph, L – larva; nd – not developed; type of seta: S – smooth; B – barbed.

Materials are deposited in collections of the University Museum Bergen and Department of Evolutionary Biology, Faculty of Biological Sciences, Kazimierz Wielki University.

A total of 21 females, 10 males, 12 deutonymphs, 8 protonymphs and 10 larvae of *Z. forsslundi* were studied. Mites were extracted using Tullgren funnels for 7 days and mounted in PVA mounting medium (Lactic Acid, Poly Vinyl Acetate, and Phenol Solution, BioQuip Products, Inc., CA, USA). The drawings were made using a Nikon Eclipse E200 microscope equipped with a Nikon Y-IDT drawing tube, and then edited with Corel Draw 2017. Measurements and transmitted-light photomicrographs were made using a Leica DM3000 equipped with a Leica DFC420 camera and Leica Application Suite 3.8. For scanning electron microscopy (SEM), the mites were air-dried, sputter coated with Au in an Agar Scientific AGB7340, and placed on Al-stubs with double-sided adhesive tape. Observations and SEM micrographs were made with a Thermo Fisher Scientific Phenom Pure microscope. All measurements are given in micrometres (μm). The terminology follows Lindquist and Evans^12^ and Lindquist and Moraza^13^ for idiosomal setation and Johnston and Moraza^14^ for the notation of dermal glands and lyrifissures. The setal terminology and notation of dermal glands and lyrifissures used in the descriptions of other Zerconidae species discussed in this paper have been converted to the above-mentioned systems if necessary.

## Results

### Morphological ontogeny of *Zercon forsslundi* (Figs. 1, 2, 3, 4, 5, 6, 7, 8, 9, 10, 11, 12, 13, 14, 15, 16)

**Fig. 1:**
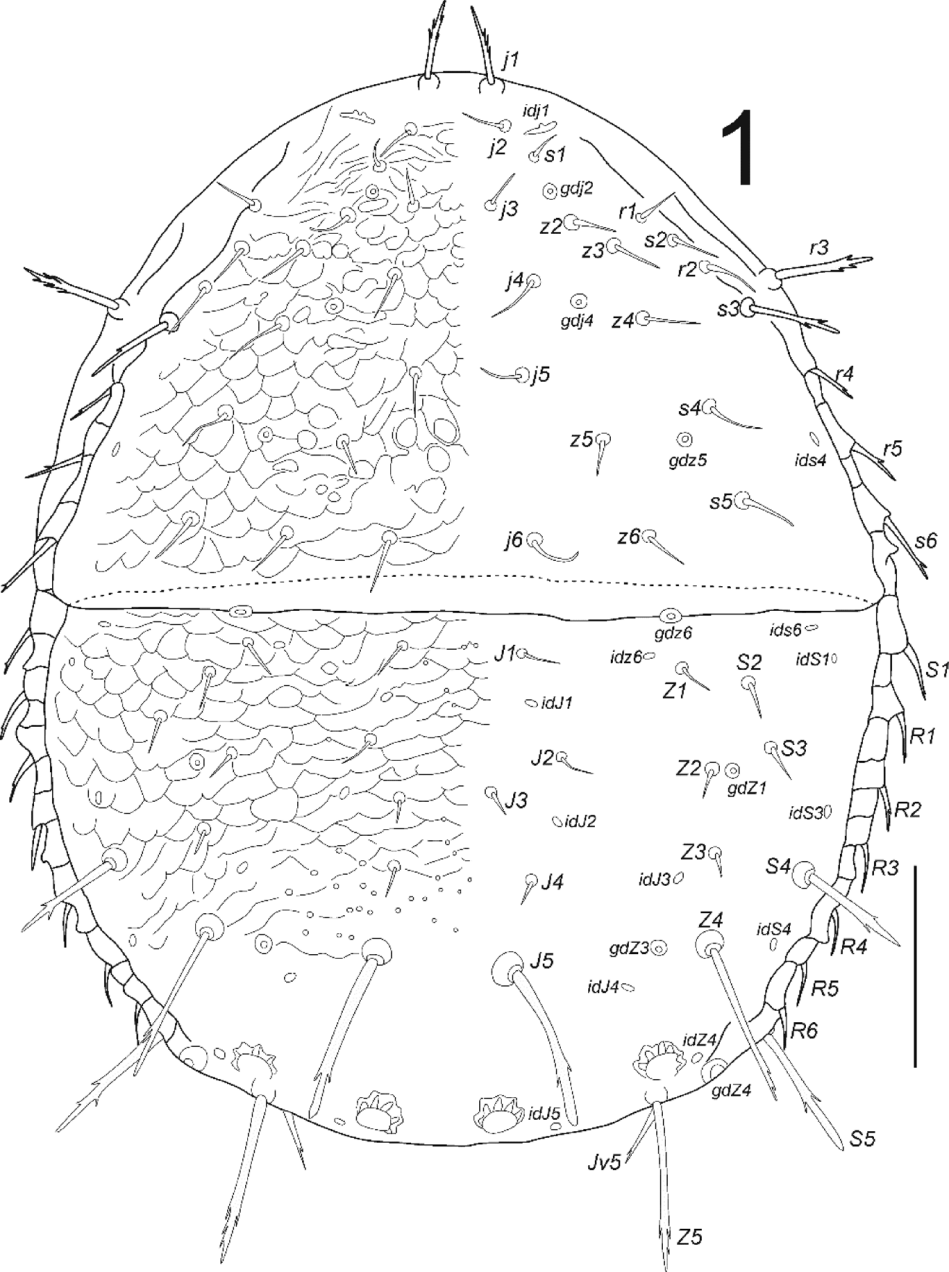
*Zercon forsslundi*, female. Dorsal aspect. Scale bar: 100 µm.

**Fig. 2:**
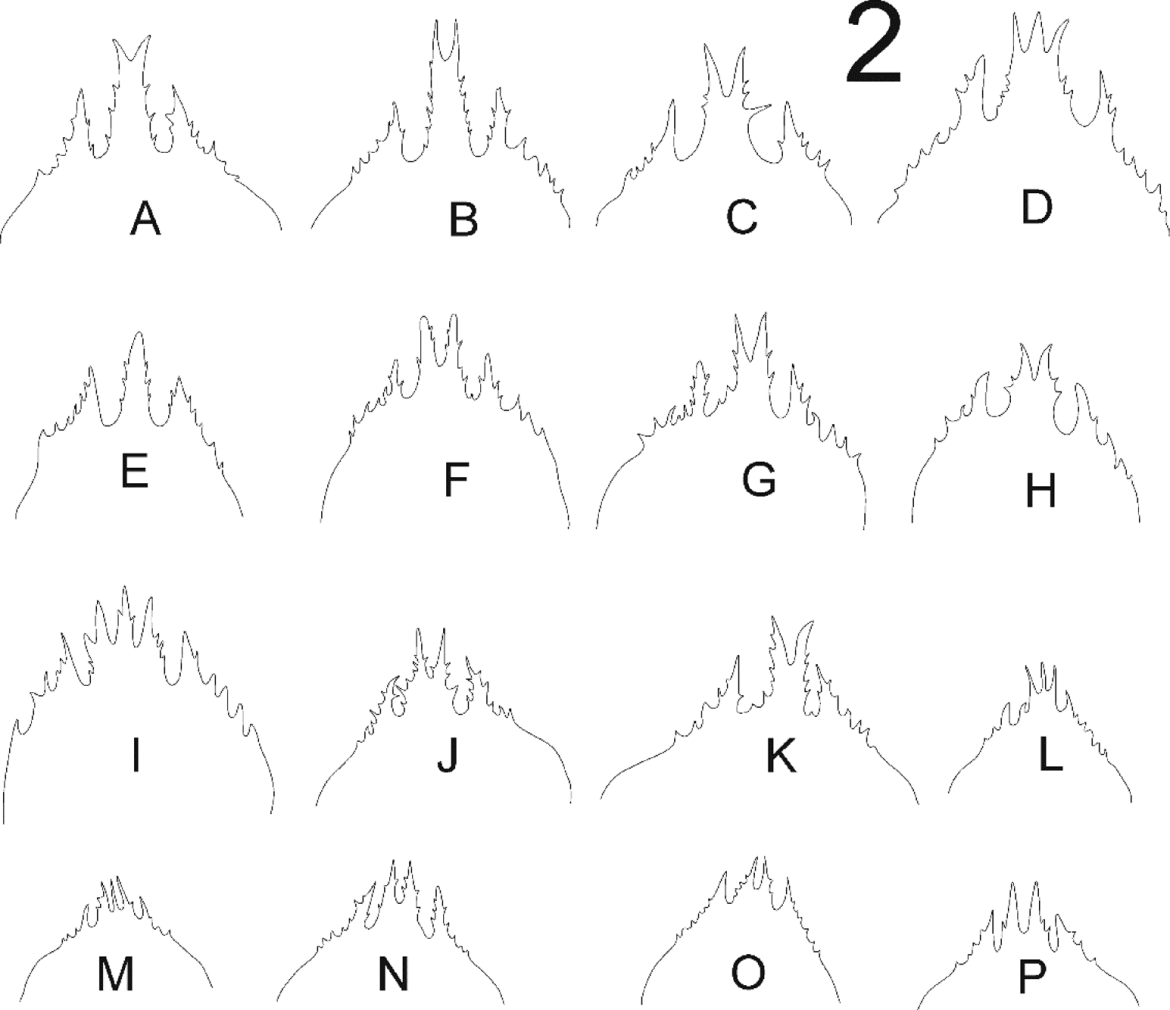
*Zercon forsslundi*, epistomes. (A–D) female; (E–I) male; (J–L) deutonymph; (M–O) protonymph; (P) larva.

**Fig. 3:**
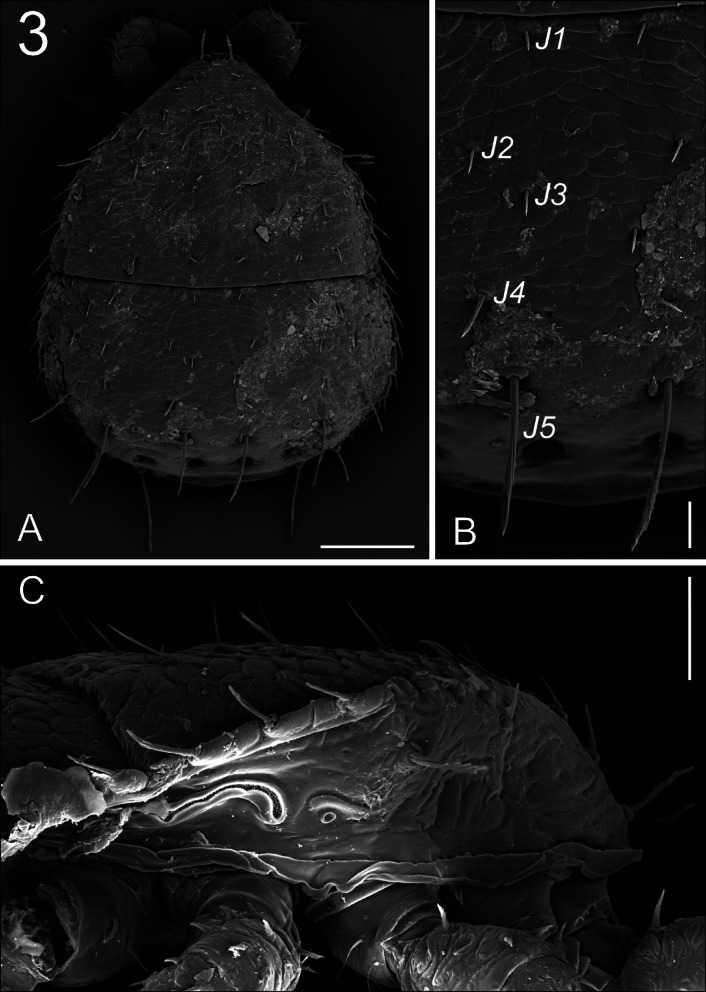
*Zercon forsslundi*, female. SEM micrographs. (A) female dorsal aspect; (B) setae of *J*-series; (C) lateral view of peritrematal area. Scale bars: (A) 100 µm; (B) 20 µm; (C) 40 µm.

**Fig. 4:**
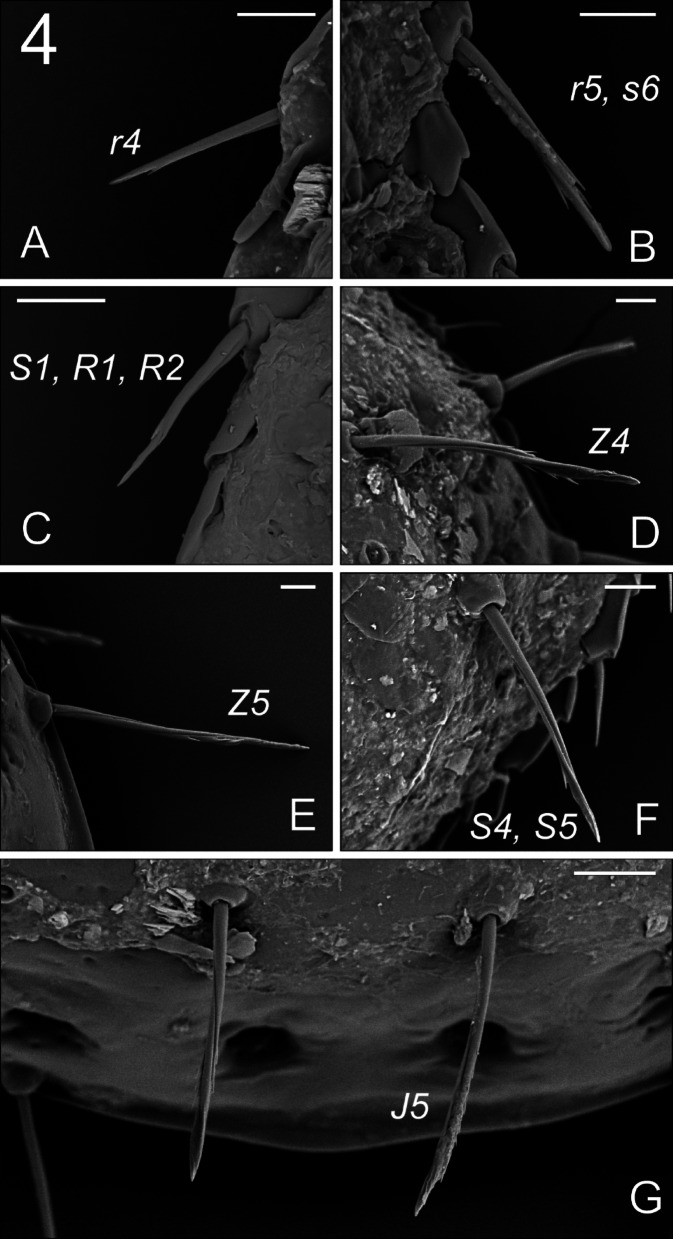
*Zercon forsslundi*, female. SEM micrographs. Character of dorsal setae: (A) *r4*; (B) *r5* and *s6*; (C) *S1*, *R1* and *R2*; (D) *Z4*; (E) *Z5*; (F) *S4* and *S5*; (G) *J5*. Scale bars: (A–F) 10 µm; (G) 20 µm.

**Fig. 5:**
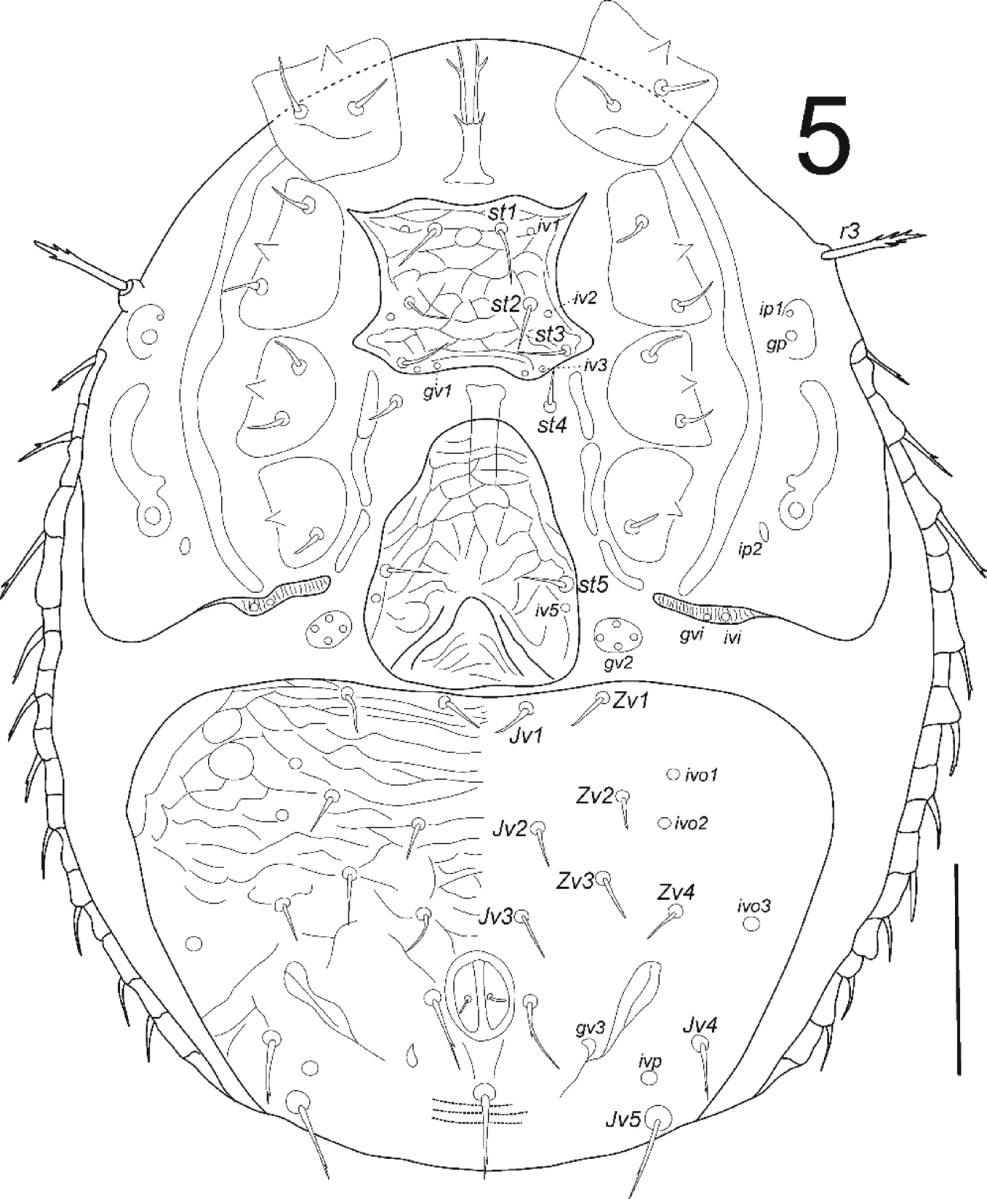
*Zercon forsslundi*, female. Ventral aspect. Scale bar: 100 µm.

**Fig. 6:**
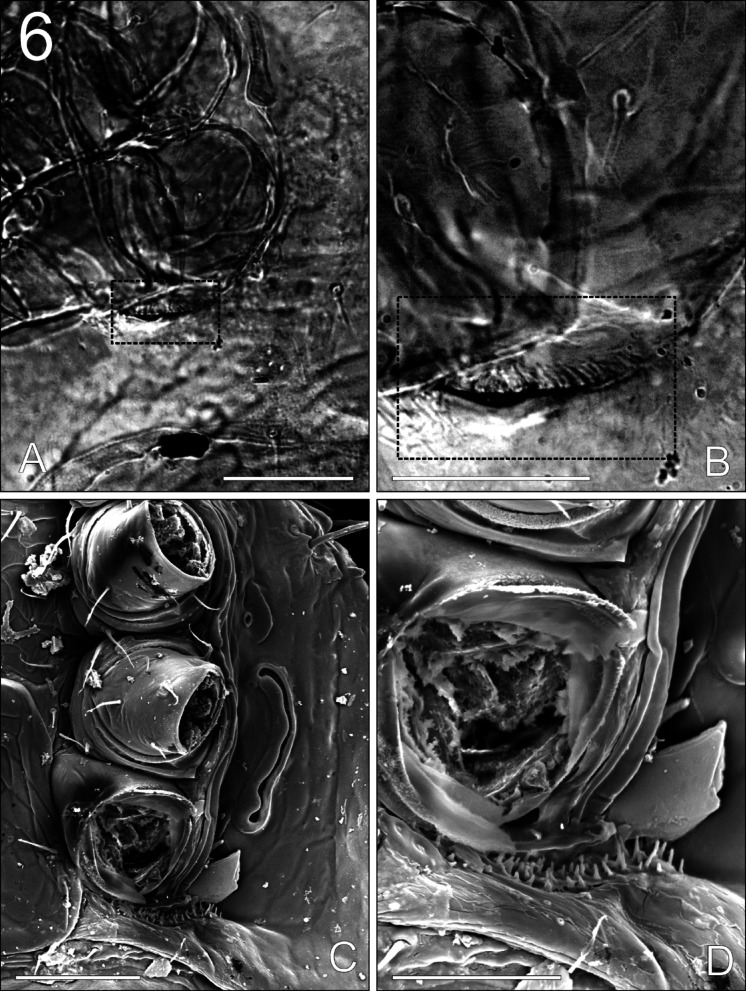
*Zercon forsslundi*, female. Inguinal region: (A, B) light microscopy (dashed squares enclose the same region at different magnifications); (C, D) scanning electron microscopy. Scale bars: (A, C) 50 µm; (B, D) 30 µm.

**Fig. 7:**
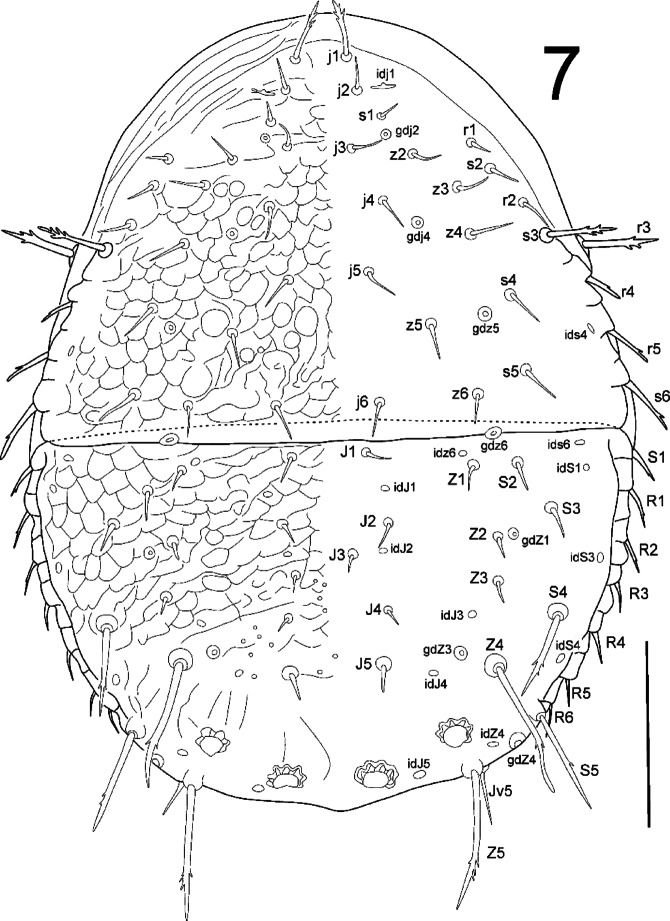
*Zercon forsslundi*, male. Dorsal aspect. Scale bar: 100 µm.

**Fig. 8:**
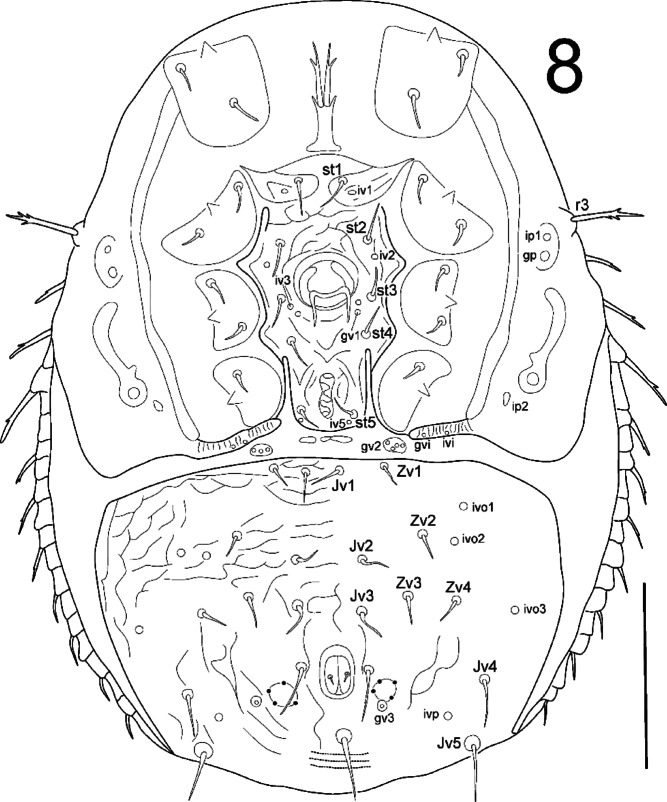
*Zercon forsslundi*, male. Ventral aspect. Scale bar: 100 µm.

**Fig. 9:**
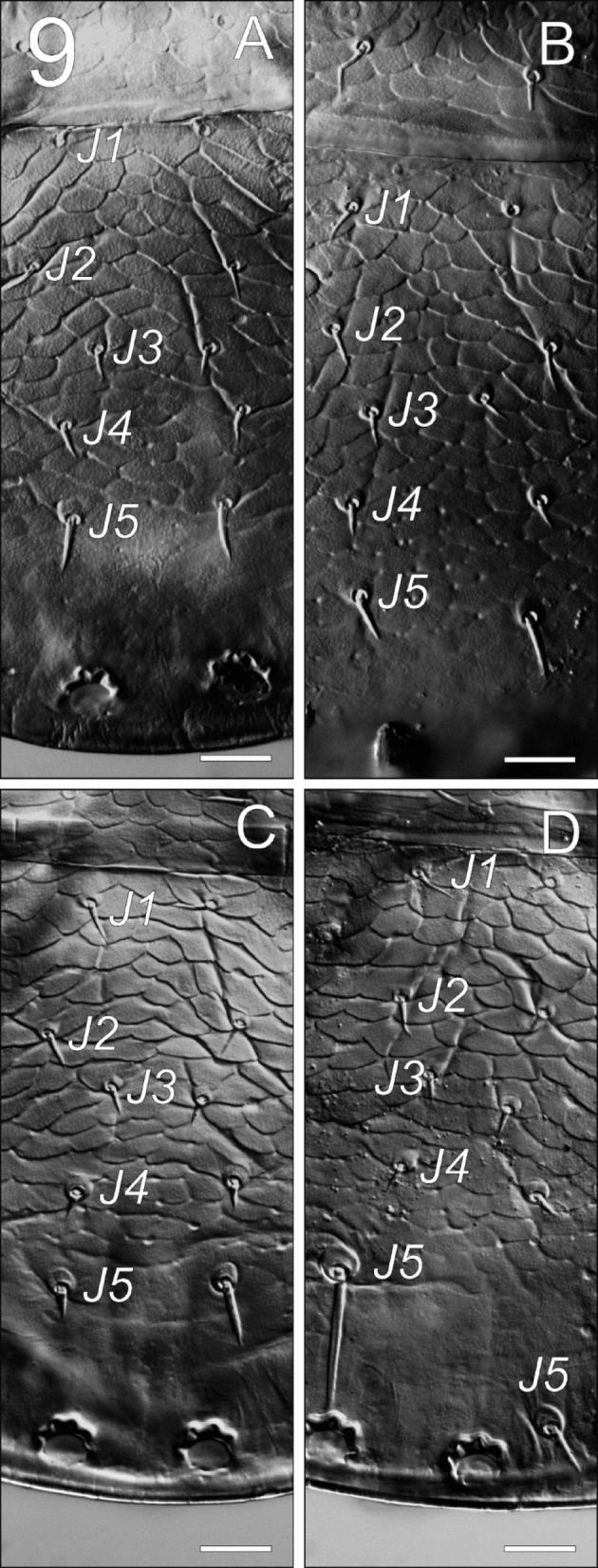
*Zercon forsslundi*, unusual opisthonotal chaetotaxy in male. (A, B) both *J5* longer than usual (intermediate length); (C) left *J5* of typical length, right seta longer (intermediate length); (D) left *J5* very long, right seta longer than usual (intermediate length) and displaced posteriorly. Scale bars: 20 µm.

**Fig. 10:**
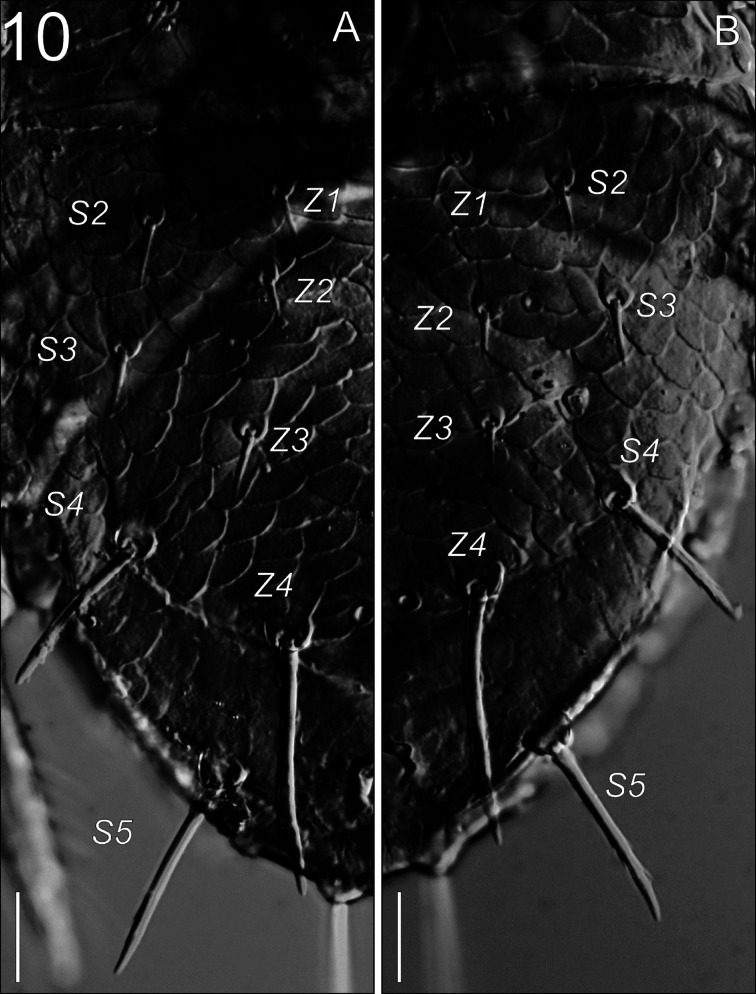
*Zercon forsslundi*, unusual opisthonotal chaetotaxy in male. (A) left *Z3* longer than usual; (B) right *Z3* of typical length (both pictures of the same individual). Scale bars: 20 µm.

**Fig. 11:**
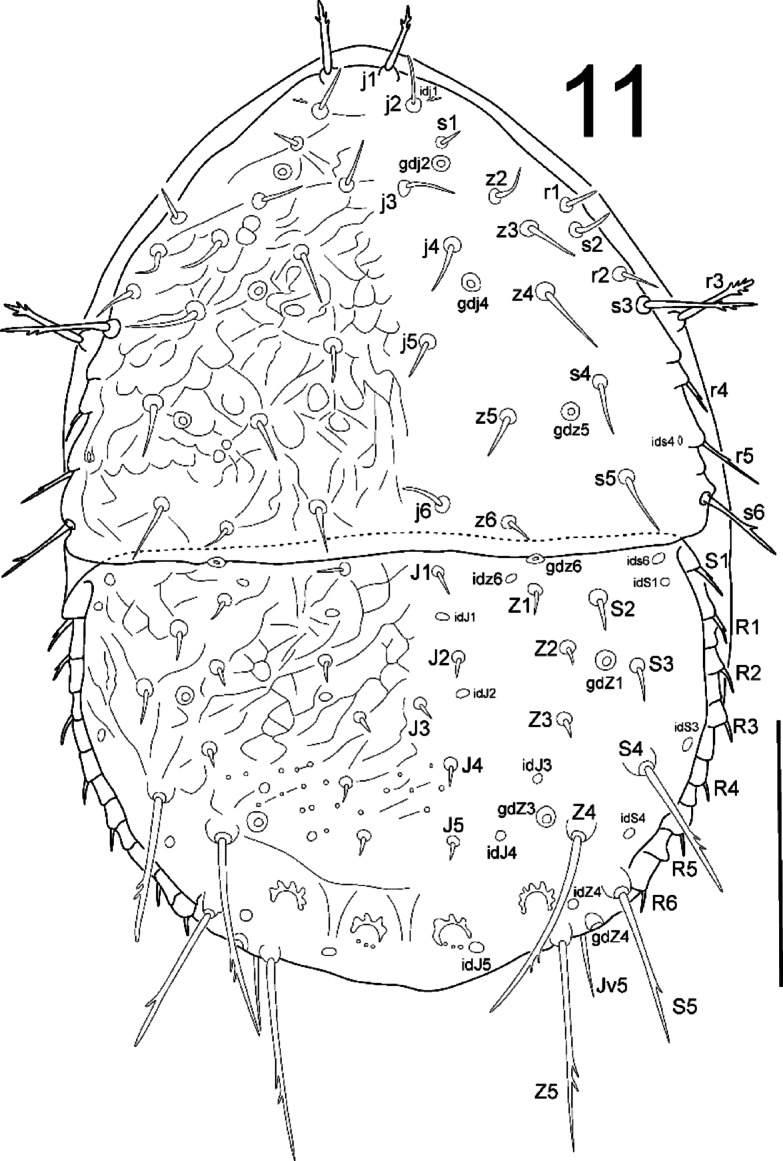
*Zercon forsslundi*, deutonymph. Dorsal aspect. Scale bar: 100 µm.

**Fig. 12:**
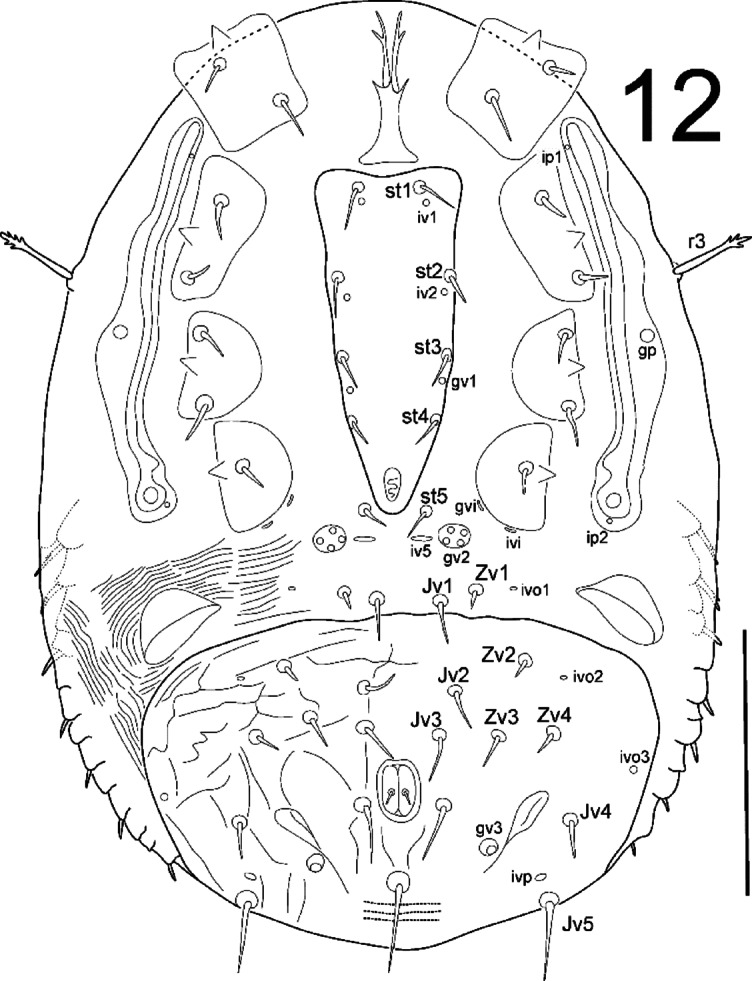
*Zercon forsslundi*, deutonymph. Ventral aspect. Scale bar: 100 µm.

**Fig. 13:**
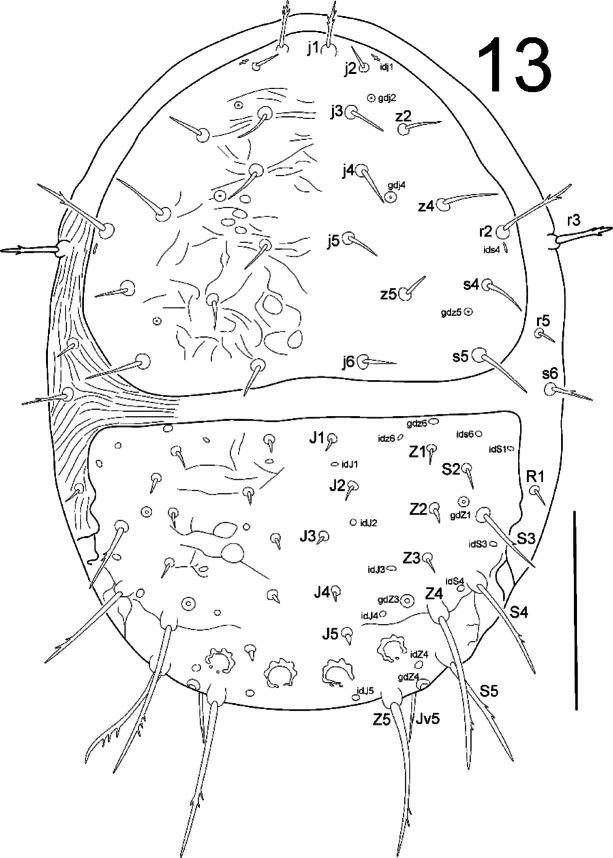
*Zercon forsslundi*, protonymph. Dorsal aspect. Scale bar: 100 µm.

**Fig. 14:**
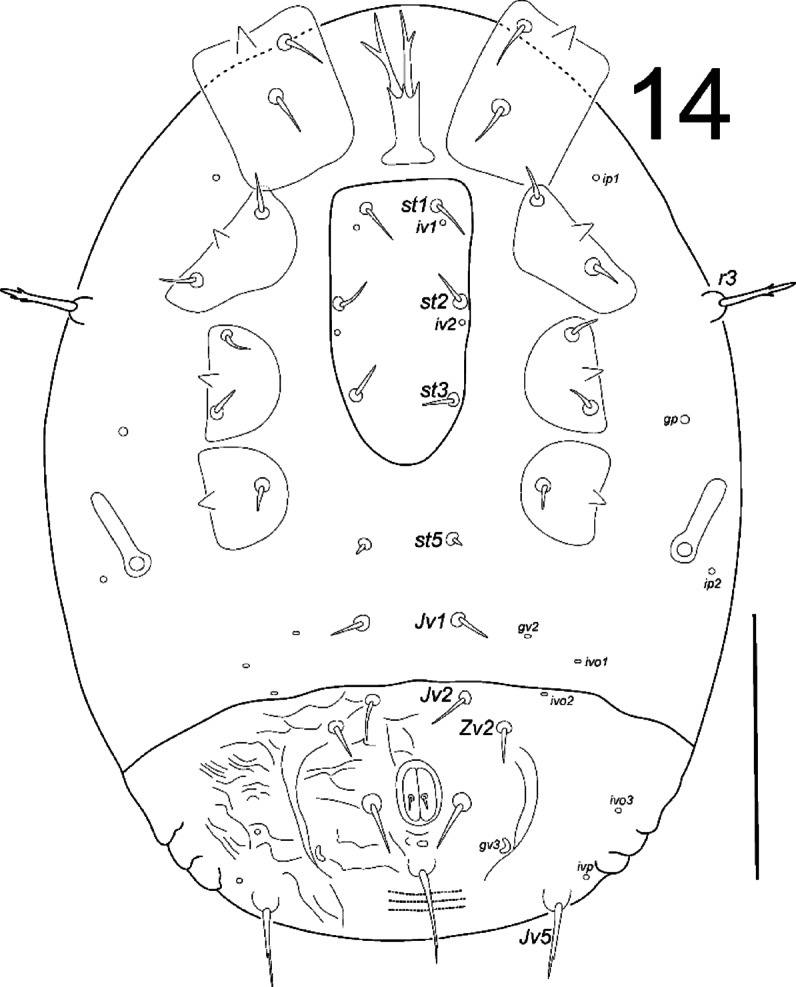
*Zercon forsslundi*, protonymph. Ventral aspect. Scale bar: 100 µm.

**Fig. 15:**
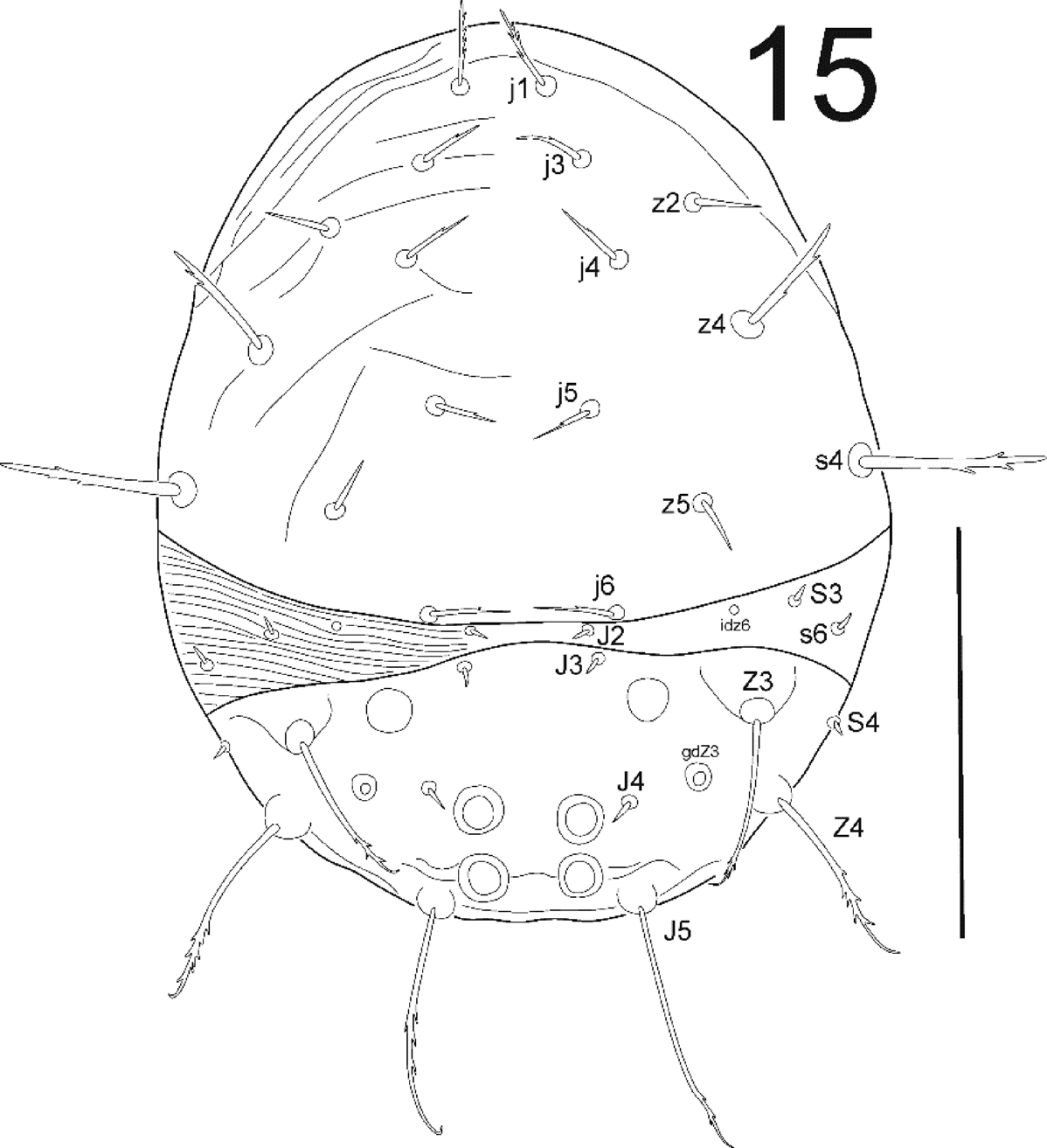
*Zercon forsslundi*, larva. Dorsal aspect. Scale bar: 100 µm.

**Fig. 16:**
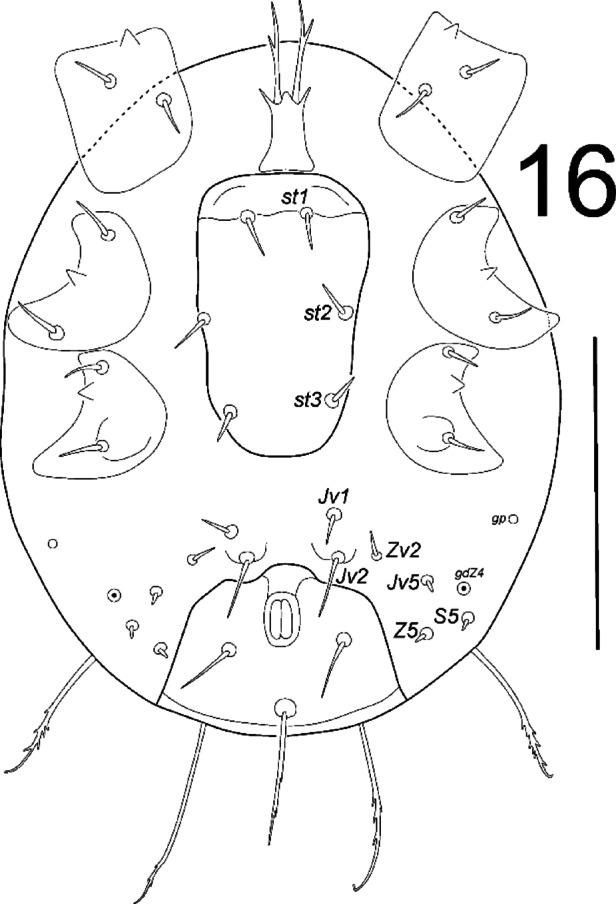
*Zercon forsslundi*, larva. Ventral aspect. Scale bar: 100 µm.

- Superorder: Parasitiformes Reuter, 1909
- Order: Mesostigmata G. Canestrini, 1891
- Family: Zerconidae G. Canestrini, 1891
- *Zercon forsslundi* Sellnick, 1958

#### Diagnosis (adult female)

Anterior margin of ventrianal shield with two pairs of setae (*Zv1* present). Pores *gv3* located posteriorly to line connecting para-anal setae close to line connecting setae *Jv4*. Podonotal shield with 21 pairs of setae, covered entirely with irregular tile-like sculpture. Setae *j1*, *s3* and *r4*–*r5,* and *s6* barbed. Seta *r3* barbed, located on margin of idiosoma. Opisthonotal shield with 21 pairs of setae (*Z2* present). All opisthonotal setae without apical hyaline endings. Setae *J1*–*J4,* *Z1*–*Z3,* and *S2–S3* short, smooth and pointed, never reach the insertions of next setae in series and never extend beyond the edge of opisthonotum. Seta *S3* similar in length to *S2*, smooth and pointed. Other opisthonotal setae long and barbed. Seta *J5* never reach beyond posterior edge of opisthonotum, but reach posterodorsal cavities. Pores *gdZ1* (*Po2*) close and posterior to line connecting insertions of setae *Z2* and *S3*, closer to *Z2*. Pores *gdZ3* (*Po3*) close to the line connecting the insertions of setae *J5* and *Z4*, closer to *Z4*. Pores *gdZ3* and insertions of setae *J5* and *Z4* located distinctly anterior to posterodorsal cavities.

#### Description

##### Female (n = 13)

Idiosoma length 524–572, width (at level of anterior edge of ventrianal shield) 410–445.

*Dorsal idiosoma* (Figs. 1, 3A,B, 4). Podonotal shield with 21 pairs of setae; all without hyaline endings. Lengths of podonotal setae of females summarized in Table 1. Seta *j1* longest in *j*-series, other *j*-series setae of comparable length. Setae *j1* with subapical barbs. Setae *j2*–*j6* smooth and pointed. All *z*-series setae of comparable length, smooth and pointed. Seta *s1* shortest of the *s*-series. Setae *s3* and *s6* of comparable length and longest of *s*-series. Seta *s3* with subapical barbs, seta *s6* with one subapical barb, other *s*-series setae smooth and pointed. Setae *r1* and *r2* smooth and pointed, setae *r4*–*r5* with one subapical barb. Seta *r1* shortest and *r5* longest of *r*-series on podonotal shield. Podonotal shield covered entirely with irregular tile-like sculpture. Location of podonotal glands: *gdj2* (*po1*) posterior to insertion of seta *s1*, adaxial and usually clearly closer to line connecting setae *s1* and *z2*; *gdj4* (*po2*) always close and posterior to the line connecting insertions of setae *j4* and *z4,* usually equally distant from insertions of *j4* and *z4* (if not then slightly moved towards *j4*); *gdz5* (*po3*) always posterior to line connecting insertions of setae *z5* and *s4*, always closer to *s4*. Location of podonotal poroids: *idj1* always abaxial to *j2* and anterior to *s1*; *ids4* close to lateral margin of podonotal shield, close to the level of insertion of seta *r5*; *idj3*, *idj6* and *idz4* not visible. Opisthonotal shield with 21 pairs of setae; all without hyaline endings. Lengths of opisthonotal setae and longitudinal distances between insertions of setae in specific series of females summarized in Table 2. Setae *J1*–*J4* short, smooth and pointed. Seta *J5* clearly longer, with subapical barbs. Seta *J5* never reach beyond posterior edge of opisthonotum and clearly reach posterodorsal cavities. Setae *Z1*–*Z3* of comparable length, short, smooth and pointed. Setae *Z4* and *Z5* clearly longer, with subapical barbs. Seta *Z4* clearly reach beyond lateral edge of opisthonotum, and almost reach the insertion of *Z5*. Seta *Z5* slightly longer (1.1 times) than *Z4*. Distance between pair of setae *Z5* 184–202. Setae *S2* and *S3* of comparable length and shortest of *S*-series, smooth and pointed. Setae *S1* with one subapical barb, *S4* with subapical barbs. Setae *S2* and *S3* never reach the insertion of next seta in series. Seta *S4* clearly longer (2.5–3.2 times) than *S2* and *S3*. Seta *S5* with subapical barbs, longer (1.1–1.3 times) than *S4*. Setae *R1*–*R6* of similar length (17–30), pointed, *R1* and *R2* with subapical barb. Opisthonotal shield covered with tile-like structure from the anterior edge to *J4*–*S4* line. The area between lines *J4***–***J4* and *J5***–***J5* with irregular lines and some bright spots in the axial part. Remaining posterior surface of opisthonotum without sculpture, finely dotted. Posterodorsal cavities crescent-like with undulating anterior margins. Location of opisthonotal glands: *gdz6* (*Po1*) anterior to *Z1* (abaxial or adaxial to *Z1*); *gdZ1* (*Po2*) always close and posterior to line connecting insertions of setae *Z2* and *S3*, always closer to insertion of *Z2*; *gdZ3* (*Po3*) close to the line connecting insertions of setae *J5* and *Z4* (usually on the line but sometimes also slightly above or below), always closer to insertion of *Z4*; *gdZ4* (*Po4*) always between the insertions of setae *S5* and *Z5*; *gdJ3* not visible. Location of opisthonotal poroids: *idz6* close to insertion of *Z1* and *gdz6* (usually adaxial, rarely on the line *Z1*–*gdz6*); *idJ1* close to the line connecting insertions of setae *J1* and *J2* (usually abaxial, sometimes adaxial or on the line); *idJ2* always abaxial to line connecting insertions of setae *J2* and *J4*, on the level of insertion of *J3*; *idJ3* always posterior to line connecting insertions of setae *J4* and *Z3*, always closer to *Z3*; *idJ4* always posterior to line connecting insertions of setae *J5* and *Z4*, always adaxial to *gdZ3*; *idJ5* always close and abaxial to inner posterodorsal cavities; *idZ4* always between *gdZ4* and outer dorsal cavity; *ids6* always close to antero-abaxial corner of opisthonotal shield, close to anterior edge of shield; *idS1* postero-abaxial to *ids6*, close to lateral edge of opisthonotal shield; *idS3* single, abaxial to line connecting setae *S3* and *S4*, close to level of insertion of J3; *idS4* postero-adaxial to seta *S4*, at the level of setae *Z4*; *idR3* rarely visible, postero-adaxial to *idS3*, at the level of insertion of *R3*.

**Table 2:** Length (mean ± SD/range in µm) and smoothness of opisthonotal setae with longitudinal distances between insertions of setae in females, males and juveniles of *Zercon forsslundi*.

	♀♀		♂♂		D		P		L	
	*Z. forsslundi*									
	Length	Type	Length	Type	Length	Type	Length	Type	Length	Type
*J1*	18 ± 1/17–20	S	15 ± 1/14–17	S	9 ± 1/8–10	S	7 ± 1/6–9	S	nd	–
*J1–J2*	52 ± 4/43–60		40 ± 2/36–44		38 ± 2/34–40		31 ± 2/27–34		n/m	
*J2*	17 ± 1/16–18	S	15 ± 2/13–19	S	7 ± 1/6–8	S	7 ± 1/6–9	S	5 ± 0.4/4–5	S
*J2–J3*	39 ± 4/33–44		28 ± 3/24–34		27 ± 3/23–33		27 ± 4/22–35		n/m	
*J3*	13 ± 1/11–15	S	11 ± 1/10–12	S	7 ± 1/6–8	S	6 ± 1/5–8	S	5 ± 0.4/5–6	S
*J3–J4*	39 ± 5/29–48		33 ± 5/24–43		30 ± 5/26–40		34 ± 5/24–40		n/m	
*J4*	13 ± 1/12–16	S	11 ± 1/10–13	S	7 ± 1/6–8	S	6 ± 1/5–7	S	6 ± 0.4/6–7	S
*J4–J5*	47 ± 5/35–54		31 ± 4/24–36		28 ± 2/23–31		27 ± 3/22–32		n/m	
*J5*	80 ± 3/74–85	B	12 ± 2/10–14	S	8 ± 1/7–8	S	6 ± 1/4–7	S	70 ± 6/60–79	B
*Z1*	18 ± 1/17–20	S	16 ± 2/11–20	S	9 ± 1/8–12	S	8 ± 1/6–11	S	nd	–
*Z1–Z2*	51 ± 7/36–64		37 ± 7/22–47		35 ± 5/25–41		34 ± 3/32–40		n/a	
*Z2*	16 ± 1/14–18	S	14 ± 2/11–17	S	8 ± 1/7–9	S	7 ± 1/6–9	S	nd	–
*Z2–Z3*	41 ± 5/32–58		35 ± 7/27–51		26 ± 3/23–30		25 ± 2/23–29		n/a	
*Z3*	14 ± 1/11–16	S	13 ± 2/9–16	S	7 ± 1/5–8	S	7 ± 1/6–8	S	52 ± 4/41–58	B
*Z3–Z4*	57 ± 7/46–69		46 ± 4/39–54		39 ± 2/35–42		36 ± 2/32–39		n/m	
*Z4*	80 ± 3/70–85	B	71 ± 3/66–74	B	78 ± 2/75–82	B	81 ± 4/73–89	B	62 ± 6/49–67	B
*Z4–Z5*	91 ± 7/70–105		61 ± 5/56–71		55 ± 3/51–60		47 ± 4/41–54		n/m	
*Z5*	87 ± 2/84–90	B	76 ± 4/69–81	B	82 ± 4/77–90	B	84 ± 4/77–92	B	5 ± 1/4–6	S
*S1*	25 ± 1/24–27	B	20 ± 2/18–24	B	16 ± 2/13–18	B	nd	–	nd	–
*S1–S2*	78 ± 5/65–87		51 ± 9/37–66		43 ± 3/39–48		n/a		n/a	
*S2*	20 ± 1/19–22	S	18 ± 1/16–20	S	12 ± 1/10–13	S	11 ± 1/8–14	S	nd	–
*S2–S3*	39 ± 4/31–45		33 ± 3/26–39		28 ± 3/24–33		26 ± 2/24–32		n/a	
*S3*	19 ± 1/17–21	S	18 ± 2/15–20	S	13 ± 1/10–15	S	43 ± 3/35–49	B	5 ± 0.4/4–5	S
*S3–S4*	69 ± 5/58–77		50 ± 6/38–58		44 ± 4/39–50		38 ± 3/36–44		n/m	
*S4*	56 ± 2/53–60	B	48 ± 2/45–50	B	57 ± 2/54–61	B	57 ± 3/52–61	B	4 ± 0.3/4–5	S
*S4–S5*	86 ± 4/78–94		63 ± 3/58–67		55 ± 4/49–61		46 ± 2/44–51		n/m	
*S5*	68 ± 1/65–71	B	57 ± 1/53–59	B	62 ± 2/57–66	B	63 ± 4/58–69	B	5 ± 0.6/4–6	S

D – deutonymph, P – protonymph, L – larva; nd – not developed, n/a – not available, n/m – not measured; type of seta: S – smooth; B – barbed.

*Gnathosoma*. Epistome (Fig. 2A–D) typically shaped for genus, with medial process bifurcated. One female with medial process tripartite.

*Ventral idiosoma* (Fig. 5). Epigynal and peritrematal shields shaped typically for genus. The peritrematal groove is directed laterally, with fine and dense pilae inside (Fig. 3C). Seta *r3* with subapical barbs. Sternal shield fully covered with reticulate ornamentation, setae *st1*–*st5* similar in length, 15–27, smooth and pointed. Ventrianal shield length 204–225, width (at level of *Jv2* setae) 315–333, with 21 setae; *Jv4*, *Jv5*, para-anal and postanal setae with single subapical barb. Ventrianal shield covered with barely reticulate ornamentation in the anterior part (to level of *Jv3* setae), remaining posterior surface of ventrianal shield without reticulate ornamentation, finely dotted. Setae *Jv1*, *Jv2*, *Zv1*–*Zv4* 15–23, *Jv3* 20–30, *Jv4* 25–30, para-anal setae 31–38, *Jv5* and postanal setae 31–40. Three parallel lines of denser dots visible posterior to postanal seta (length of each line does not exceed distance between para-anal setae). Anus length 21–25, width 17–21. Location of ventral glands: *gv1* close to insertion of *st3*, *gv2* multiple and typically located; *gvi* in the inguinal region; *gv3* (posterior para-anal glands) posterior to line connecting para-anal setae, close to line connecting setae *Jv4*; *gp* anterior to peritreme. Location of ventral poroids: *iv1* abaxial to insertion of *st1*; *iv2* postero-abaxial to insertion of *st2*; *iv3* close to the posterior edge of sternal shield, adaxial to insertion of *st3*; *iv5* on the genital shield, posterior to insertion of seta *st5*; *ip1* anterior to *gp*; *ip2* postero-adaxial to stigma; *ivo1* and *ivo2* located at level of *Jv2*–*Zv2* setae (usually *ivo1* anterior to *ivo2*, rarely on the one side of the body *ivo1* abaxial to *ivo2*); *ivo3* close to lateral edge of ventrianal shield, close and posterior to line connecting insertions of setae *Zv4*; *ivo4* not visible; *ivi* in the inguinal region; *ivp* single, anterior to line connecting setae *Jv5*, close to insertion of *Jv5*. The inguinal region has clearly visible, dense cuticular spines directed ventro-anterally (towards the coxae) (Fig. 6).

##### Male (n = 6)

Idiosoma length 398–445, width (at level of anterior edge of ventrianal shield) 303–330.

*Dorsal idiosoma* (Figs. 7, 9, 10). Podonotal shield with 21 pairs of setae; all without hyaline endings. Lengths of podonotal setae of males summarized in Table 1. Seta *j1* longest of *j*-series, other *j*-series setae of comparable length. Seta *j1* with subapical barbs. Setae *j2*–*j6* smooth and pointed. All *z*-series setae smooth and pointed, *z2* and *z6* of comparable length and slightly shorter than *z3*–*z5*. Seta *s1* shortest of the *s*-series. Setae *s3* and *s6* of comparable length and longest of *s*-series. Seta *s3* with subapical barbs, seta *s6* with one subapical barb, other *s*-series setae smooth and pointed. Setae *r1* and *r2* smooth and pointed, setae *r4* and *r5* with one subapical barb. Seta *r1* shortest and *r5* longest of *r*-series on podonotal shield. Podonotal shield covered entirely with irregular tile-like sculpture. Location of podonotal glands: same as in female. Location of podonotal poroids: same as in female. Opisthonotal shield with 21 pairs of setae; all without hyaline endings. Lengths of opisthonotal setae and longitudinal distances between insertions of setae in specific series of males summarized in Table 2. Setae *J1*–*J5* short, smooth and pointed. Seta *J5* clearly thickened when compared to *J1*–*J4*. In addition, we have observed four males with *J5* setae longer than usual: two males with both *J5* setae longer and thicker than in other males, but still considerably shorter than those in females (16–19, intermediate length, barbed); one male with one of the *J5* setae short (10, typical length, smooth) and the other longer (21, intermediate length, barbed); one male with one of the *J5* considerably longer than usual in males (58, resembling the female’s *J5*, barbed), and the other of intermediate length (18, barbed) (Fig. 9A–D). Setae of *Z*-series similar to those in female. Seta *Z4* clearly extends beyond lateral edge of opisthonotum, and insertion of *Z5*. Seta *Z5* slightly longer (1.01–1.14 times) than *Z4*. In studied males, the length of *Z3* seta ranged from 9 to 16, with both setae of comparable length in specific individuals. In one male we found unusual length of *Z3* setae; one *Z3* seta was short (12) while the other one was clearly longer (20) (Fig. 10). Distance between pair of setae *Z5* 144–156. Setae of *S*-series similar to those in female. Seta *S4* clearly longer (2.5–3.2 times) than *S3*. Seta *S5* with subapical barbs, longer (1.1–1.2 times) than *S4*. Setae *R1*–*R6* 13–23, *R1* and *R2* with one subapical barb. Opisthonotal shield covered with tile-like structure from the anterior edge to *J4*–*S4* line. The area between lines *J4*–*J4* and *J5*–*J5* with irregular lines and some bright spots in the axial part. Remaining posterior surface of opisthonotum without sculpture, finely dotted. Posterodorsal cavities crescent-like with undulating anterior margins. Location of opisthonotal glands: *gdZ1* (*Po2*) usually close and posterior to the line connecting insertions of setae *Z2* and *S3* (sometimes on the line or slightly above), always closer to insertion of *Z2*; *gdZ3* (*Po3*) usually anterior to the line connecting insertions of setae *J5* and *Z4* (sometimes on the line, never posterior), always closer to insertion of *Z4*; other glands as in female. Location of opisthonotal poroids: *idz6* close to insertion of *Z1* and *gdz6*, always adaxial to the line *Z1*–*gdz6*; *idJ1* close to the line connecting insertions of setae *J1* and *J2*, usually abaxial (rarely on the line); *idJ3* always posterior to line *J4*–*Z3*, at the level of insertions of *J4* and *S4*, often closer to *Z3*, rarely equally distant to *J4* and *Z3*; other poroids similar as in female.

*Gnathosoma*. Epistome (Fig. 2E–I) typically shaped for genus with medial process bifurcated. One male with medial process tripartite, the other one male with undivided medial process.

*Ventral idiosoma* (Fig. 8). Genital and peritrematal shields shaped typically for genus. Seta *r3* with subapical barbs. Sternal shield covered with irregular ornamentation. Sternal setae smooth and pointed, *st1*–*st5* 13–21 long. Ventrianal shield length 164–177, width (at level of *Jv2* setae) 250–268, with 21 setae. Para-anal setae with one subapical barb, other setae smooth. Ventrianal shield covered with barely reticulate ornamentation in the anterior part (to level of *Jv3* setae), remaining posterior surface of ventrianal shield without reticulate ornamentation, finely dotted. Setae *Jv1*, *Jv2*, *Zv1*–*Zv4* 10–19, *Jv3* 18–23, *Jv4* 21–26, para-anal setae 23–30, *Jv5* and postanal setae 28–35. Three parallel lines of denser dots visible posterior to postanal seta (length of each line does not exceed distance between para-anal setae). Anus length 18–20, width 15–16. Location of ventral glands: *gv1* adaxial to line connecting insertions of setae *st3* and *st4*; location of other glands same as in female. Location of ventral poroids: *iv1* postero-abaxial to insertion of *st1*; *iv3* postero-adaxial to insertion of *st3*; *iv5* on the sternal shield, posterior to insertion of seta *st5*; location of other poroids same as in female.

##### Deutonymph (n = 6)

Idiosoma length 358–414, width (at level of anterior edge of ventrianal shield) 264–314.

*Dorsal idiosoma* (Fig. 11). Podonotal shield with 21 pairs of setae; all without hyaline endings. Lengths of podonotal setae of deutonymphs summarized in Table 1. Seta *j1* longest of *j*-series, other *j*-series setae of comparable length. Seta *j1* with subapical barbs, other *j*-series setae smooth and pointed. All *z*-series setae smooth and pointed, *z6* shortest and *z4* longest. Seta *s1* shortest of *s*-series. Setae *s2* slightly longer than *s1*, *s4* and *s5* clearly longer and of comparable length. Setae *s3* and *s6* of comparable length and longest of *s*-series. Seta *s3* with subapical barbs, seta *s6* with one subapical barb, other *s*-series setae smooth and pointed. Setae *r1* and *r2* smooth and pointed, setae *r4*–*r5* with one subapical barb. Seta *r1* shortest and *r5* longest of *r*-series on podonotal shield. Podonotal shield covered with irregular net-like sculpture. Location of podonotal glands and poroids: same as in male and female. Opisthonotal shield with 21 pairs of setae; all without hyaline endings. Lengths of opisthonotal setae and longitudinal distances between insertions of setae in specific series of deutonymphs summarized in Table 2. Setae *J1*–*J5* as in male: short, smooth and pointed; seta *J5* slightly thickened when compared to *J1*–*J4*. Setae of *Z*-series similar to those in female. Seta *Z4* clearly extends beyond lateral edge of opisthonotum, and insertion of *Z5*. Setae *Z4* and *Z5* of comparable length. Distance between pair of setae *Z5* 114–124. Seta *S1* with one subapical barb, *S4* with subapical barbs. Setae *S2* and *S3* of comparable length and shortest of *S*-series, smooth and pointed, never reach the insertion of next seta in series. Seta *S4* clearly longer (3.9–5.8 times) than *S2* and *S3*. Seta *S5* with one subapical barb, slightly longer (1.02–1.16 times) than *S4*. Setae *R1*–*R6* of similar length (7–15), pointed, *R1* with one subapical barb. Opisthonotal shield covered with irregular net-like sculpture from the anterior edge to *J4*–*S4* line. The area between lines *J4–J4* and *J5*–*J5* with irregular lines and some bright spots in the axial part, remaining posterior surface of opisthonotum without sculpture, finely dotted. Posterodorsal cavities crescent-like with undulating anterior margins. Location of opisthonotal glands: *gdZ1* (*Po2*) often close and posterior to line *Z2*–*S3* (sometimes on the line; rarely slightly anterior), often equally distanced to *Z2* and *S3*, sometimes clearly closer to *Z2*; *gdZ3* (*Po3*) close and always anterior to the line connecting insertions of setae *J5* and *Z4*, always closer to insertion of *Z4*; other opisthonotal glands same as in male and female. Location of opisthonotal poroids: *idJ4* close to line *J5*–*Z4* (usually on the line, rarely posterior), always adaxial to *gdZ3*; *idz6* and *idJ3* same as in male; *idJ1* same as in female; *idR3* not visible.

*Gnathosoma*. Epistome (Fig. 2J–L) typically shaped for genus. One deutonymph with medial process tripartite.

*Ventral idiosoma* (Fig. 12). Seta *r3* with subapical barbs. Sternal shield without visible ornamentation, finely dotted. Setae *st1*–*st5* 8–17, smooth and pointed (*st5* off the shield). Ventrianal shield length 130–148, width (at level of *Jv3* setae) 195–213, with 17 pointed setae (*Jv1* and *Zv1* off the shield). Postanal seta with subapical barb, other setae smooth. Ventrianal shield covered with barely visible ornamentation and finely dotted. Setae *Jv1*–*Jv4* and *Zv3* 11–19, *Zv1*, *Zv2* and *Zv4* 7–10, para-anal setae 21–23, and postanal seta 33–44 long. Three parallel lines of denser dots visible posterior to postanal seta (length of each line does not exceed distance between para-anal setae). Anus length 16–17, width 13–15. Location of ventral glands: *gv1* between insertions of setae *st3* and *st4*; *gv2* multiple and typically located; *gv3* (posterior para-anal glands) posterior to line connecting para-anal setae, posterior to line connecting setae *Jv4*; *gp* close to peritreme, at the level of coxa III; *gvi* close to posterior edge of coxa IV. Location of ventral poroids: *iv1*–*iv2* posterior to insertions of *st1* and *st2* respectively; *iv3* not visible; *iv5* posterior to insertion of seta *st5*, at the level of *gv2*; *ip1* at the level of anterior edge of coxa II; *ip2* similar to those of adults; *ivo1* always outside the ventrianal shield, at the level of *Zv1*; *ivo2* always on the shield, posterior to level of *Zv2* and antero-abaxial to *Zv4*; *ivo3* close to lateral edge of ventrianal shield, close to line adjacent to anterior edge of anus; *ivo4* not visible; *ivp* single, anterior to line connecting setae *Jv5*, close to insertion of *Jv5*; *ivi* close to posterior edge of coxa IV. Soft cuticular area between ventral shields with visible parallel corrugations.

##### Protonymph (n = 5)

Idiosoma length 360–390, width (at level of anterior edge of opisthonotal shield) 273–310.

*Dorsal idiosoma* (Fig. 13). Podonotal shield with 12 pairs of setae; all without hyaline endings. Lengths of podonotal setae of protonymphs summarized in Table 1. Seta *j1* longest of *j*-series, *j2* shortest, other *j*-series setae of comparable length. Seta *j1* with subapical barbs, other *j*-series setae smooth and pointed. Seta *z4* longest of *z*-series, other *z*-series setae of comparable length, all *z*-setae smooth and pointed. Setae of *s*-series of comparable length. Seta *s6* with one subapical barb and off the podonotal shield, other *s*-setae smooth. Seta *r2* on the podonotal shield, longest on podonotal shield, with subapical barbs. Seta *r5* off the podonotal shield, short, smooth and pointed. Podonotal shield covered with irregular lines (delicately corrugated). Location of podonotal glands: *gdj2* (*po1*) postero-abaxial to insertion of seta *j2*, adaxial and close to line connecting insertions of setae *j2* and *z2*; other podonotal gland same as in female and male. Location of podonotal poroids: *idj1* antero-abaxial to insertion of *j2*; *ids4* antero-abaxial to insertion of seta *s4*, posterior to insertion of seta *r2*. Soft cuticular area between dorsal shields with visible parallel corrugations. Opisthonotal shield with 14 pairs of setae; all without hyaline endings. Lengths of opisthonotal setae and longitudinal distances between insertions of setae in specific series of protonymphs summarized in Table 2. Setae *J1*–*J5* short, smooth and pointed. Seta *J5* visibly thicker when compared to *J1*–*J4*. Setae *Z1*–*Z3* short, smooth and pointed. Setae *Z4* and *Z5* clearly longer, with barbs located at about one third or one fourth from seta termination. Seta *Z4* clearly reach beyond lateral edge of opisthonotum, and reach beyond insertion of *Z5*. Setae *Z4* and *Z5* of similar length. Distance between pair of *Z5* setae 93–101. Seta *S2* shortest of *S*-series, smooth and pointed. Setae *S3*–*S5* longer with subapical barbs. Seta *S3* clearly longer (2.9–4.4 times) than *S2*. Seta *S2* never reach insertion of *S3*. Setae *S3*–*S4* clearly reach the insertions of next setae in series. Setae *S4* longer (1.2–1.5 times) than *S3*. Setae *S4* and *S5* more similar in length (*S5* 1.02–1.2 times longer than *S4*). Seta *R1* off the opisthonotal shield, short (8–10), smooth and pointed. Opisthonotal shield covered with barely visible corrugations between *J-* and *Z-*series. Remaining posterior surface of opisthonotum finely dotted. Posterodorsal cavities with undulated anterior margin; outer cavities crescent-like, inner ones more rounded. Location of opisthonotal glands: *gdZ1* (*Po2*) usually anterior to insertions of setae *Z2* and *S3*, usually slightly closer to insertion of *S3*; *gdZ3* (*Po3*) always anterior to line *J5*–*Z4*, always closer to insertion of seta *Z4*; other glands same as in adults and deutonymph. Location of opisthonotal poroids: *idJ1* close to line connecting insertions of setae *J1* and *J2*, usually on the line (rarely adaxial to the line); *idJ3* close to line (usually anterior) connecting insertions of setae *J4* and *Z3*; *idJ4* always anterior to line connecting insertions of setae *J5* and *Z4*, always adaxial to *gdZ3*; *idS3* single, usually slightly adaxial to line connecting insertions of setae *S3* and *S4*, at the level of insertions of *J3*; *idS4* close and postero-adaxial to insertion of *S4; idz6* same as in males and deutonymphs; other poroids as in adults and deutonymphs; *idR3* not visible.

*Gnathosoma*. Epistome (Fig. 2M–O) typically shaped for genus.

*Ventral idiosoma* (Fig. 14). Setae *r3* with subapical barbs. Sternal shield without ornamentation. Setae *st1*–*st3* 11–15, *st5* off the sternal shield and shorter 4–6. Ventrianal shield with 9 smooth and pointed setae (*Jv1* off the shield), with weak ornamentation (corrugations). Setae *Jv1*, *Jv2* and *Zv2* of comparable length (11–16), para-anal setae longer 19–21, postanal seta 34–36. Seta *Jv5* 26–33 long with subapical barb. Three parallel lines of denser dots visible posterior to postanal seta (length of each line does not exceed distance between para-anal setae). Anus length 15–20, width 12–13. Location of ventral glands: *gv2* single, abaxial to *Jv1* setae, close to the line connecting insertions of these setae; *gv3* (posterior para-anal glands) posterior to line connecting para-anal setae; *gp* anterior to short peritreme, at the level of posterior edge of coxa III. Location of ventral poroids: *ip1* at the level of anterior edge of coxa II; *ip2* postero-abaxial to stigma; *iv1* posterior to insertion of *st1*; *iv2* posterior to insertion of *st2*; *ivo1* always outside the ventrianal shield, postero-abaxial to *gv2*; *ivo2* usually on the anterior edge of the shield at the level of *Jv2*, sometimes outside the shield; *ivo3* close to lateral edge of ventrianal shield, at the level of para-anal setae; *ivo4* not visible; *ivp* single, antero-abaxial to insertion of seta *Jv5*. Soft cuticular area between ventral shields with visible parallel corrugations.

##### Larva (n = 7)

Idiosoma length 220–290, width (at level of setae *S3*) 186–226.

*Dorsal idiosoma* (Fig. 15). Podonotal shield with nine pairs of setae; all without hyaline endings. Lengths of podonotal setae of larvae summarized in Table 1. All *j*-series setae of comparable length, *j1* with subapical barbs, *j2***–***j6* with one subapical barb. Setae *z2* and *z5* short, smooth and of comparable length, seta *z4* clearly longer, with subapical barbs. Seta *s4* longest of podonotal shield, with subapical barbs. Seta *s6* off the shield, diminutive. Location of podonotal glands: *gdj4* and *gdz5* not visible. Location of podonotal poroids: *idj3*, *idj6*, *idz4* and *ids4* not visible. Soft cuticular area between dorsal shields with visible parallel corrugations. Opisthonotal shield with 6 pairs of setae; all without hyaline endings. Lengths of opisthonotal setae in larvae summarized in Table 2. Setae *J2* and *S3* off shield, diminutive. Setae *J3*, *J4* and *S4* similar in length, diminutive, smooth and pointed. Setae *Z3*, *Z4* and *J5* with subapical barbs and usually curved terminations. Seta *Z4* longer (1.1–1.3 times) than *Z3*. Seta *J5* longest of opisthonotal shield, longer (1.04–1.31 times) than *Z4*. Location of opisthonotal glands: *gdJ3* not visible; *gdZ3* (*Po3*) always close and posterior to line connecting insertions of setae *J4* and *Z3*, usually equally distant from their insertions; sometimes slightly closer to insertion of *Z3*, rarely closer to insertion of *J4*. Location of opisthonotal poroids: *idz6* close to line connecting insertions of setae *S3*, abaxial to insertion of *J2*; *idJ3*, *idJ5* and *idR3* not visible. Podonotal and opisthonotal shields with irregular pattern, delicately corrugated. Soft cuticular area between dorsal shields with visible parallel corrugations. Posterodorsal cavities circular with smooth edges, arranged typically for the genus.

*Gnathosoma.* Epistome (Fig. 2P) typically shaped for genus.

*Ventral idiosoma* (Fig. 16). Sternal shield without ornamentation. Setae *st1*–*st3* 10–16. Ventrianal shield subtriangular, bell-shaped (not measured), with three setae (*Jv1*, *Jv2*, *Jv5* and *Zv2* off the shield). Setae *Jv5* 4–6, *Jv1* and *Zv2* 7–12, *Jv2* 19–24, para-anal setae 19–26 and postanal seta 42–51 long. Anal opening poorly visible (not measured). Setae *Z5* and *S5* diminutive, abaxial to ventrianal shield, at the level of para-anal setae. Location of ventral glands: *gdZ4* (*Po4*) anterior to line connecting insertions of para-anal setae, abaxial to line connecting insertions of setae *Jv5* and *S5*; *gp* close to lateral edge of the idiosoma, at the level of setae *Jv1* and *Zv2*; *gv3* not visible. Location of ventral poroids: *ivo4* and *ivp* not visible. Soft cuticular area between ventral shields with visible parallel corrugations.

### Distribution of *Zercon forsslundi*, *Z. polonicus* and *Z. hamaricus* (Fig. 17)

**Fig. 17:**
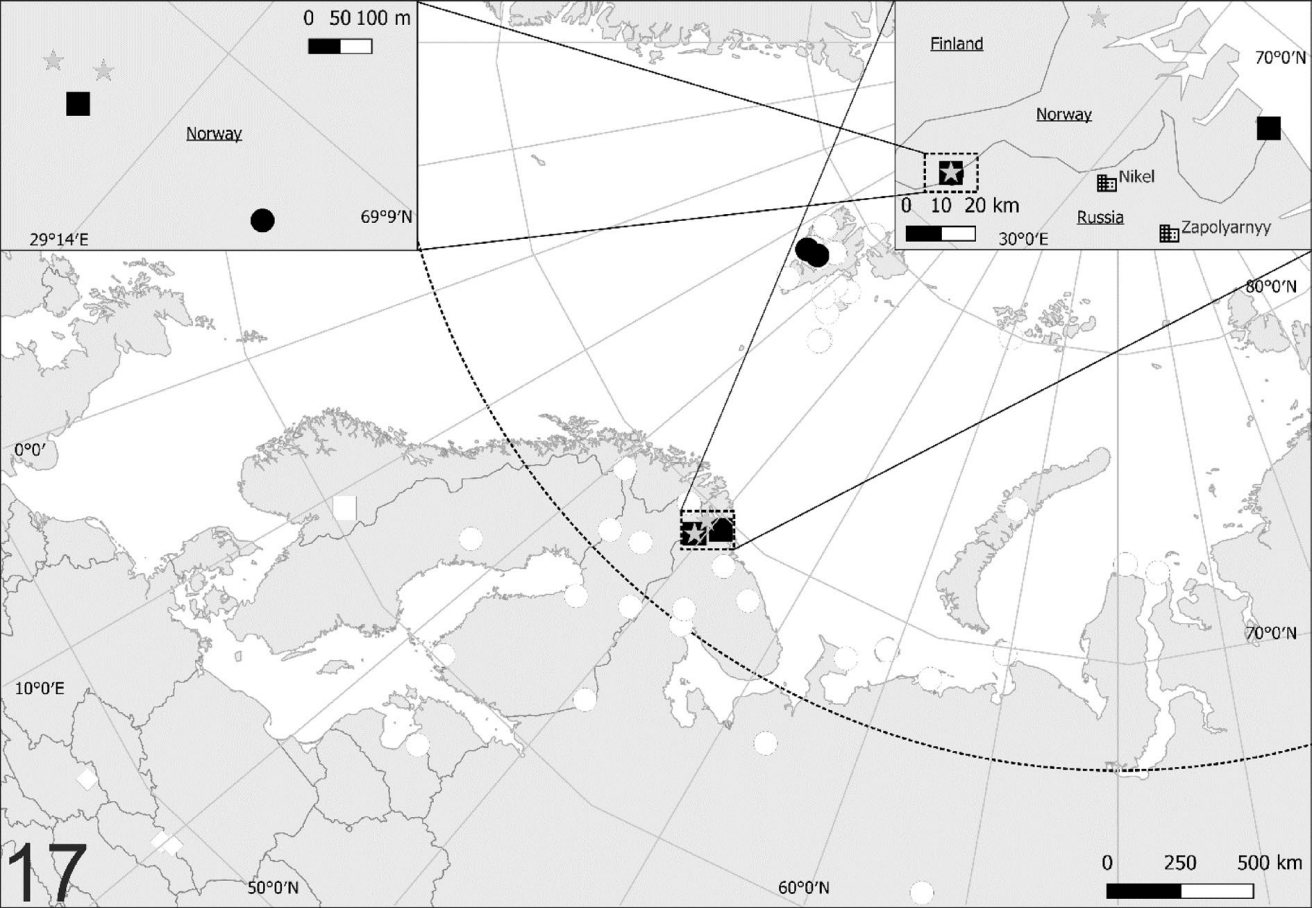
Distribution map of *Zercon forsslundi*, *Z. hamaricus* and *Z. polonicus*. Empty markers (earlier records): circles—*Z. forsslundi*; diamonds—*Z. polonicus*; squares—*Z. hamaricus*. Filled markers (new records): black circles—*Z. forsslundi*; black squares—*Z. hamaricus*; gray stars – sympatric populations of *Z. forsslundi* and *Z. hamaricus*, dotted line—Arctic Circle.

Altogether, 50 presence points of *Z. forsslundi* were used for analysis, including six new localities^4,15–35^ (for new localities see Additional material, Table A1). The majority presence points (42%) were located in Svalbard (21, including two localities used for the current description). One third of localities (15 presence points) were located in Russia, with most of them (14) in the northern part of the country (the Murmansk and Arkhangelsk Oblasts, the Nenets and Yamalo-Nenets Autonomous Okrugs), with the exception of the southernmost finding in Russia (the Pechora-Ilych Nature Reserve in Komi Republic). The other 14 presence points are located in Finland (7, throughout the country), Sweden (2, northern and central parts), Norway (4, northern part, new data) and Latvia (1, the southernmost presence point of *Z. forsslundi*). Except for the localities of *Zercon polonicus* used by Błaszak^6,36^ for the species description (S Poland, Tatra and Pieniny Mountains) it was also found in NE Austria (Saubrunn, Lower Austria)^37^. *Zercon hamaricus* was described by Kaczmarek et al.^5^ based on material from S Norway, here we proved its presence in five new localities in N Norway.

## Discussion

The newly collected adults of *Z. forsslundi* agree very well with the description of Sellnick^4^; however, we found some minor differences (Table 3) that widens our knowledge of the intraspecific variability of the studied species. The most pronounced are: (1) the placement of dorsal cavities that in our individuals are relatively close to the posterior edge of opisthonotum when compared with drawings of Sellnick^4^; (2) insertions of *J5* and *Z4* according to Sellnick’s description are on the same transverse line, while in most of our individuals the line connecting insertions of these setae is arcuate. Sellnick described setae *Z4*, *Z5*, *S4* and *S5* as distally flattened; terminations of these setae are just like that; however, the SEM micrographs show, that each of these setae possesses twisted sutures running from the insertion and almost reach the distal tip of seta, which is also true for other long opisthonotal setae (Fig. 4).

**Table 3:** Comparison of characters of *Z. forsslundi* according to Sellnick^4^ with results of the present study. Measurements in µm.

Character	Sellnick’s (1958) male/female	Our data male/female
Length of *J5*	short/70	10**–**14/74**–**85
Length of *Z5*	76/84	69**–**81/84**–**90
Distance *Z5*–*Z5*	152/176	144**–**156/184**–**202
*Z5* and *Jv5* insertions	very close to each other	
Length of *Z4*	64/76	66**–**74/70**–**85
Insertions of *J5* and *Z4*	In one transverse line	In arcuate line
Length of *S4*	44/52	45**–**50/53**–**60
Length of *S5*	52/64	53**–**59/65**–**71
Insertion of *S5*	On the edge of opisthonotum	
*Z4*, *Z5*, *S4*, *S5*	flattened distally, barbed	
*gdZ3* (*Po3*)	Close to line *Z4*–*J5*, closer to *Z4*	
Body length	390/495	398**–**445/524**–**572
Body width	288/375**–**405	303**–**330/410**–**445
Dorsal cavities	not very close to posterior edge of opisthonotum	very close to posterior edge of opisthonotum

According to Sellnick’s^4^ drawing, *r4*–*r5*–*s6* and *S1* are barbed *in Z. forsslundi*. Our present study revealed that *r4*–*r5*–*s6* and *S1–R1–R2* are barbed; therefore, the characteristics of marginal setae shows more close relation between *Z. hamaricus* and *Z. forsslundi*. It is worthy to emphasize that in *Z. hamaricus* we found that setae *S1–R6* possess one subapical barb, whereas in *Z. forsslundi* we found single barb only on *S1–R2* setae. These barbs are, however, not always visible (in both light and scanning microscopy), apparently because of the various barbs locations (dorsally, ventrally, laterally). Nevertheless, we have not observed barbs on *R3–R6* setae in *Z. forsslundi*. Therefore, the smoothness of *r4*–*r5*–*s6* and *S1*–*R1*–*R6* in *Z. hamaricus*, *Z. polonicus* and *Z. forsslundi* is as follows: all barbed in *Z. hamaricus*, all smooth in *Z. polonicus* and some setae (*r4–r5–s6–S1–R1–R2*) barbed in *Z. forsslundi*.

Kaczmarek et al.^5^ pointed out close relationship of *Z. hamaricus* to *Z. forsslundi* as well as *Z. polonicus* (the similarity between the latter species has also been emphasized in the key of Karg^38^). The morphological closeness of *Z. forsslundi*, *Z. hamaricus* and *Z. polonicus* has been confirmed in immature stages (Table 4); however, to fully analyze the similarities between these species, the supplemental description of the larvae of the latter species is needed.

**Table 4:** Distinguishing characters of immature stages of *Z. forsslundi, Z. hamaricus* and *Z. polonicus*. Measurements in µm.

	*Z. forsslundi*	*Z. hamaricus*	*Z. polonicus*
Larva	*S3* and *s6* short and of comparable length (4–5) *Z3* 41–58 *Z4* 49–67 *J5* 60–79	*S3* and *s6* short and of comparable length (5–6) *Z3* 58–61 *Z4* 73–80 *J5* 102–110	n/a
Protonymph	*Z2* and *Z3* short and of comparable length (6**–**9) *S2* (8–14), smooth, does not reach insertion of *S3*	*Z2* and *Z3* short and of comparable length (7–9) *S2* (25–31), smooth, almost reach insertion of *S3*	*Z3* smooth and clearly longer (38) than *Z2* (8) *S2* (30), smooth, almost reach insertion of S3
Deutonymph	*Z3* smooth and short (5–8) *gdZ1* on line *Z2*–*S3* *r4*–*r5*–*s6* similar to *S1*–*R1* *S2* 10**–**13 *S3* 10–15, smooth, does not reach beyond half distance to insertion of *S4*	*Z3* smooth and short (10–13) *gdZ1* on line *Z2*–*S3* *r4*–*r5*–*s6* similar to *S1*–*R1*–*R6* *S2* 20–26, smooth, *S3* 50–64, barbed, clearly reach beyond half distance to insertion of *S4*	*Z3* barbed and long (44–50) *gdZ1* on line *Z3*–*S3* *r4*–*r5*–*s6* similar to *S1*–*R1*–*R6* S2 9 *S3* 19**–**23, smooth, does not reach beyond half distance to insertion of S4

n/a—stage description not available, this table supplements data in Kaczmarek et al*.* (2021)^5^.

The glacial fluctuations throughout the Quaternary undoubtedly influenced the fauna and flora of high and even mid-latitudes in the Holarctic^39^. The harsh climatic conditions have been dramatic not only for the species not adapted to low temperatures as e.g. the mite *Labidostomma luteum* Kramer, 1897^40^ that has originated from France, and the following glaciations influenced its dispersion and range of both the parthenogenetic and bisexual populations^41–43^. In parallel, changes in glacier range also influenced the Arctic that were pushed more to the south and then dispersed to the north according to ice sheets advance and retreat, respectively. Most of the presence points of *Z. forsslundi* used in this work are located within the Barents Sea region, which is an area of biotic convergence between the Palearctic and Nearctic^44^. Dispersal throughout the continental part is relatively easy to explain in mites when compared with inhabiting the Arctic islands of the Barents Sea. Phoresy is not present in all Mesostigmata. Three distinctive types of phoretic relationships were defined in this order at the superfamily level, with only four regarded as exclusively free-living (i.e. non-phoretic) taxa, including Veigaioidea, Arctacaroidea, Epicrioidea and Zerconoidea^45^. The lack of phoresy, decreasing dispersal ability, undoubtedly influenced the immigration of these invertebrates to the Arctic islands since the last glaciation^44^. In general, the invertebrates inhabiting the Barents Sea islands are regarded as relatively young fauna (acc. to the tabula rasa hypothesis) that dispersed to Svalbard, Novaya Zemlya and Franz Josef Land after the Last Glacial Maximum (LGM), however, in situ survival hypotheses (i.e. nunatak and cryptic refugia) are also under debate^44,46^ (for a discussion of tabula rasa vs nunatak hypothesis see Brochmann et al.^47^). As zoochory is not present in Zerconidae, the other possible dispersal vectors are anemochory, hydrochory and anthropochory (i.e. wind-, water- and human-induced dispersion). Anemochory has not been previously reported in Zerconidae. The survival of some Collembola and Oribatida in seawater was defined as sufficient to allow successful dispersal from N Norway to Svalbard^48^, however, no zerconid mite species have been studied in this context. The negative effect of seawater can be decreased if driftwood is considered a dispersal agent. The rafting of organic matter from Siberian rivers, supported by ocean currents, influenced the distribution of plants and, very likely, invertebrates within the Arctic since the last glaciation^29,44,48,49^. With respect to human-induced dispersal, this is not excluded, but the former studies on soils introduced to Pyramiden (Svalbard) focused on chernozem-type soils imported from southern European Russia and Ukraine, regions that cannot be considered possible *Z. forsslundi* sources. Moreover, the *Z. forsslundi* inhabits only undisturbed natural soils at Pyramiden, which can prove the vulnerability of this species to anthropogenic disturbances^30^.

In northwestern Eurasia, the LGM ice-sheet covered Fennoscandia, Svalbard, Franz Josef Land and Novaya Zemlya up to the northwestern part of Taymyr Peninsula, with the southern ice extending much to the south in the western (i.e. European) part compared with the eastern region; however, the eastern ice extent is debatable^50,51^. The LGM ice sheet extent reconstructions suggest, that North Eurasia was not glaciated from the eastern part of the Kanin Peninsula and further east to Taymyr^52^. In parallel, most of the area of N Eurasia was covered with two main vegetation types i.e. steppe-tundra and polar-alpine deserts^53^. These areas were the continental refugia for the Arctic species that subsequently spread north after ice sheet retreat.

The more southern findings of the Arctic species of *Zercon* are undoubtedly populations of glacial relicts. For these cold-adapted taxa the present climatic conditions in southern mountainous areas are suitable for survival as the equivalent of the Arctic, which was also confirmed in e.g. *Ceratozetes spitsbergensis* (Oribatida)^54^. During the periods of south-Polish glaciation (Sanian 1 period, the equivalent of the Glacial C period during the first half of middle Pleistocene: 780–420 ka BP) the ice sheet occupied most of the Polish territory, reached the Carpathians and the Sudetes and entered mountain river valleys^55^. *Zercon polonicus*, closely related to *Z. forsslundi* and *Z. hamaricus*, so far has been recorded in Tatra Mountains and Pieniny Mountains, S Poland as well as in Saubrunn (540 m a.s.l.), NE Austria^6,36,37^. Therefore, we hypothesize that *Z. polonicus* can be considered a post-glacial relict among other species with *J5* setae considerably longer than short and similar *J1*–*J4* in females. For *Z. polonicus*, which is not present in the current Arctic fauna, it could be hypothesized that it evolved e.g. from *Z. forsslundi* or other related Arctic species (e.g. *Z. hamaricus*?) that inhabited southern Europe during glaciation, and its descendant, *Z. polonicus*, formerly considered the Carpathian endemic^36,56^, migrated between the Carpathians and Alps.

## Conclusions

Here we reported that the ranges of *Z. forsslundi* and *Z. hamaricus* overlap. Moreover, we confirmed the sympatry of these two species in the area of N Norway (Additional material, Table A1). According to current data, NW Europe could be regarded as the area of interaction at the southern edge of the range of *Z. forsslundi* and the northern edge of the range of *Z. hamaricus*. We also proved that microhabitats of these species overlap in N Norway (both species were recorded in the same samples at some locations). The interactions of taxa at the edges of biogeographical ranges are extremely interesting as they influence the local/regional biodiversity. North Eurasia is the region of interaction of the Arctic fauna and more-southern non-Arctic Palearctic fauna at the northern verge of the latter, which was also previously observed in mites^20^. This region is, therefore, a large-scale transitional zone between the Arctic and non-Arctic faunas. The influence of edge effects on the biodiversity of mites was previously observed also at the local-scale^57–59^. At the southeastern verge of the Palearctic (i.e. the Korean Peninsula), the diversity of Zerconidae is also shaped by extremely diverse environmental conditions, moreover, strengthened by isolation at the southern edge of the family range^60^.

Furthermore, genetic studies should be performed on *Z. forsslundi*, *Z. hamaricus* and *Z. polonicus* to determine the phylogenetic relationships among these Palearctic species. It could then be used to study Nearctic members of *Zercon* from the same, unique morphological group. Both would be useful for studying the speciation center(s) and dispersal processes of the whole unique group in the Holarctic.

## Electronic supplementary material

Below is the link to the electronic supplementary material.

Supplementary Material 1

## Data Availability

The datasets supporting the conclusions of this article are included within the article and its additional file.
