# Supplementary material for: Complete instars’ morphology of Zercon forsslundi Sellnick, 1958 (Parasitiformes: Mesostigmata) with notes on distribution and evolution
Source: Sci Rep. 2025 Jul 2;15:23425. doi: 10.1038/s41598-025-06732-0 (PMC12223083; doi:10.1038/s41598-025-06732-0)
Supplement: Supplementary file 1 — Supplementary Material 1 [file 41598_2025_6732_MOESM1_ESM.docx]

| collection site | latitude | longitude | habitat | collected by | collection date | species | individuals |
| --- | --- | --- | --- | --- | --- | --- | --- |
| Nyrud | 69.14300N | 29.23677E | birch forest, 61 m a.s.l. | Juho Vuolteenaho | 05.10.2023 | *Z. hamaricus* | 3F |
| Nyrud | 69.14324N | 29.23490E | birch forest, 62 m a.s.l. | Arne Fjellberg | 15.07.2021 | *Z. forsslundi* | 1F |
|  |  |  |  |  | 25.08.2021 | *Z. forsslundi* | 2F |
|  |  |  |  | Juho Vuolteenaho | 29.09.2022 | *Z. forsslundi* | 2F |
|  |  |  |  |  |  | *Z. hamaricus* | 5F, 3M, 3D |
| Nyrud | 69.14347N | 29.24536E | river shore, 54 m a.s.l. | Juho Vuolteenaho | 05.10.2023 | *Z. forsslundi* | 2F |
| Nyrud | 69.14361N | 29.23668E | birch forest, 64 m a.s.l. | Juho Vuolteenaho | 30.05.2023 | *Z. forsslundi* | 1F |
|  |  |  |  |  |  | *Z. hamaricus* | 1F, 1D |
| Ferdesbekken | 69.71472N | 29.31616E | birch forest, 60 m a.s.l. | Juho Vuolteenaho | 06.06.2023 | *Z. forsslundi* | 2F, 1M |
|  |  |  |  |  |  | *Z. hamaricus* | 1F, 2M, 1D |
| Grense Jakobselv | 69.78016N | 30.823298E | estuary shore, 1m a.s.l. | Juho Vuolteenaho | 04.10.2022 | *Z. hamaricus* | 2F, 4M |

Individuals’ labels: F – females, M – males, D – deutonymphs.
